# Supplementary figures and images for: Arterial pulsations drive oscillatory flow of CSF but not directional pumping
Source: Sci Rep. 2020 Jun 22;10:10102. doi: 10.1038/s41598-020-66887-w (PMC7308311; doi:10.1038/s41598-020-66887-w)

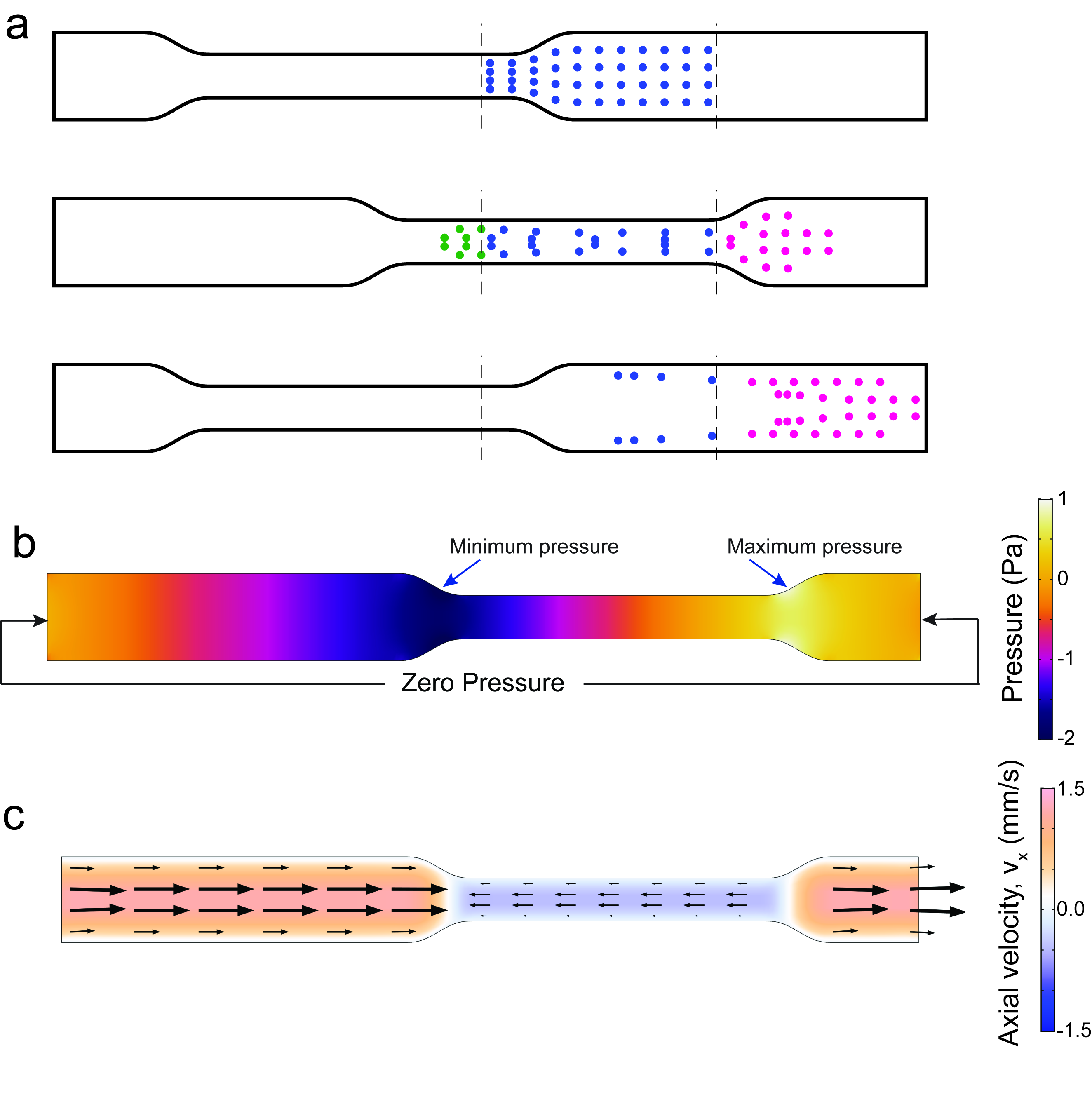

Supplement: Supplementary file 2 — Supplementary information2. [file 41598_2020_66887_MOESM2_ESM.tif]

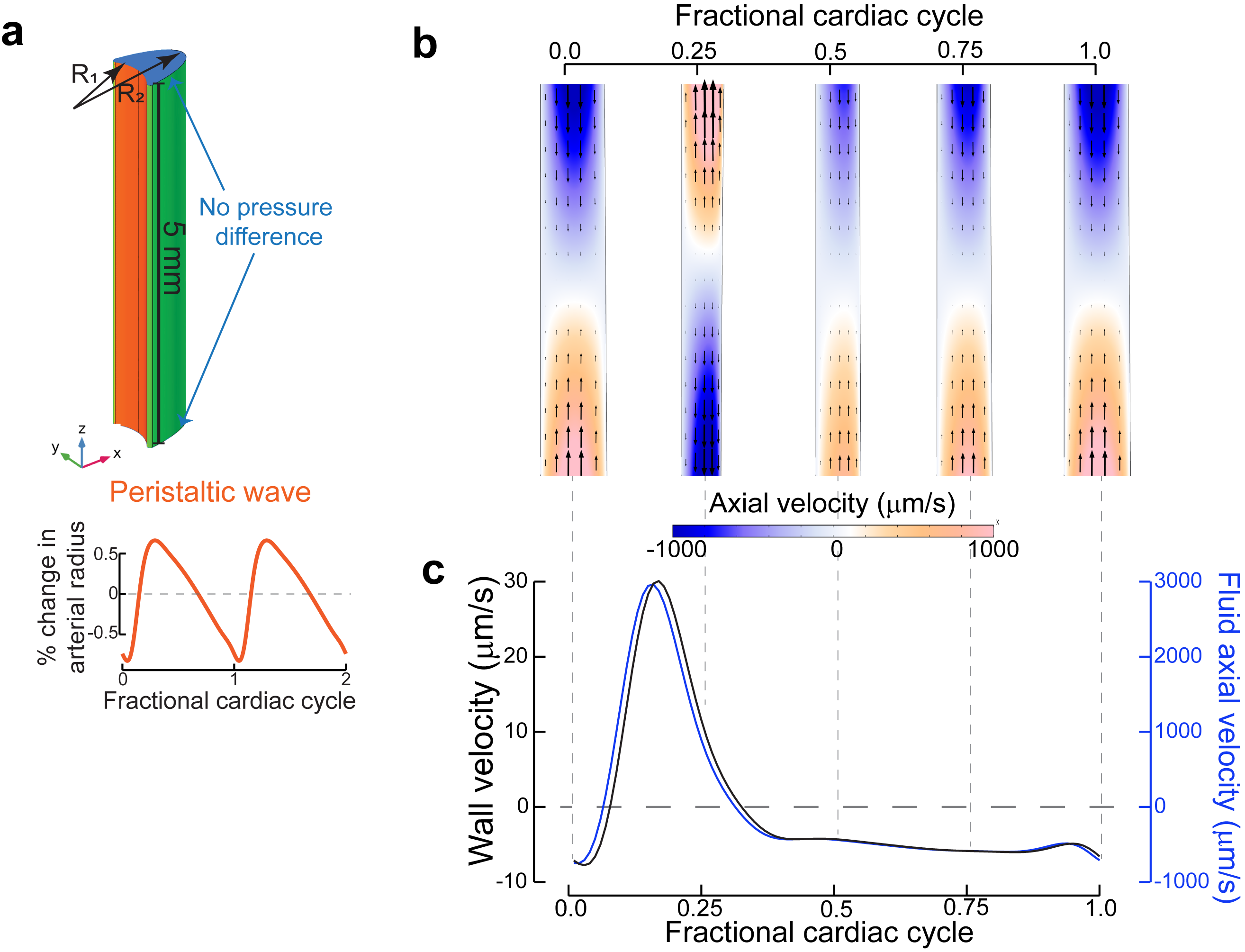

Supplement: Supplementary file 3 — Supplementary information3. [file 41598_2020_66887_MOESM3_ESM.tif]

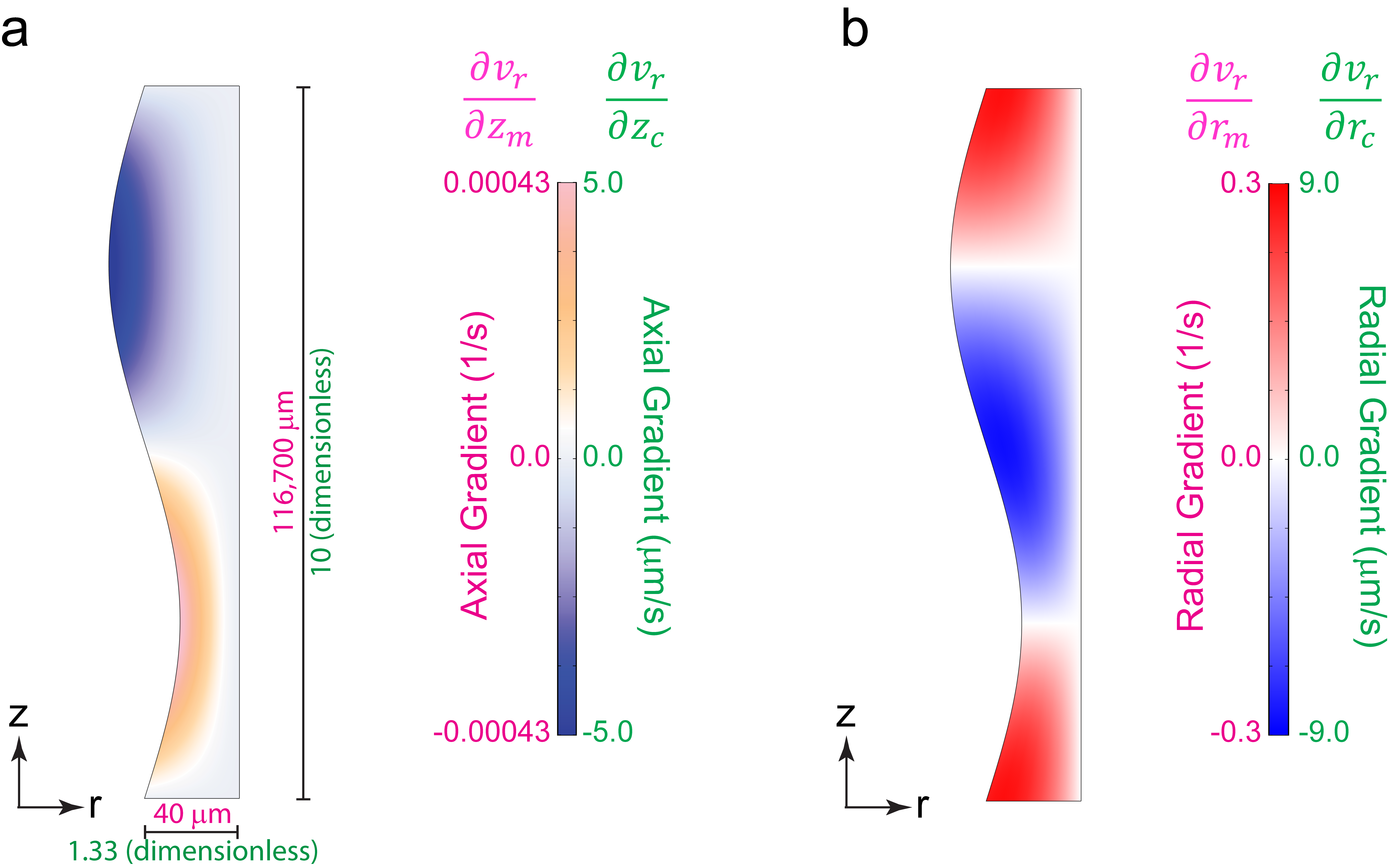

Supplement: Supplementary file 4 — Supplementary information4. [file 41598_2020_66887_MOESM4_ESM.tif]

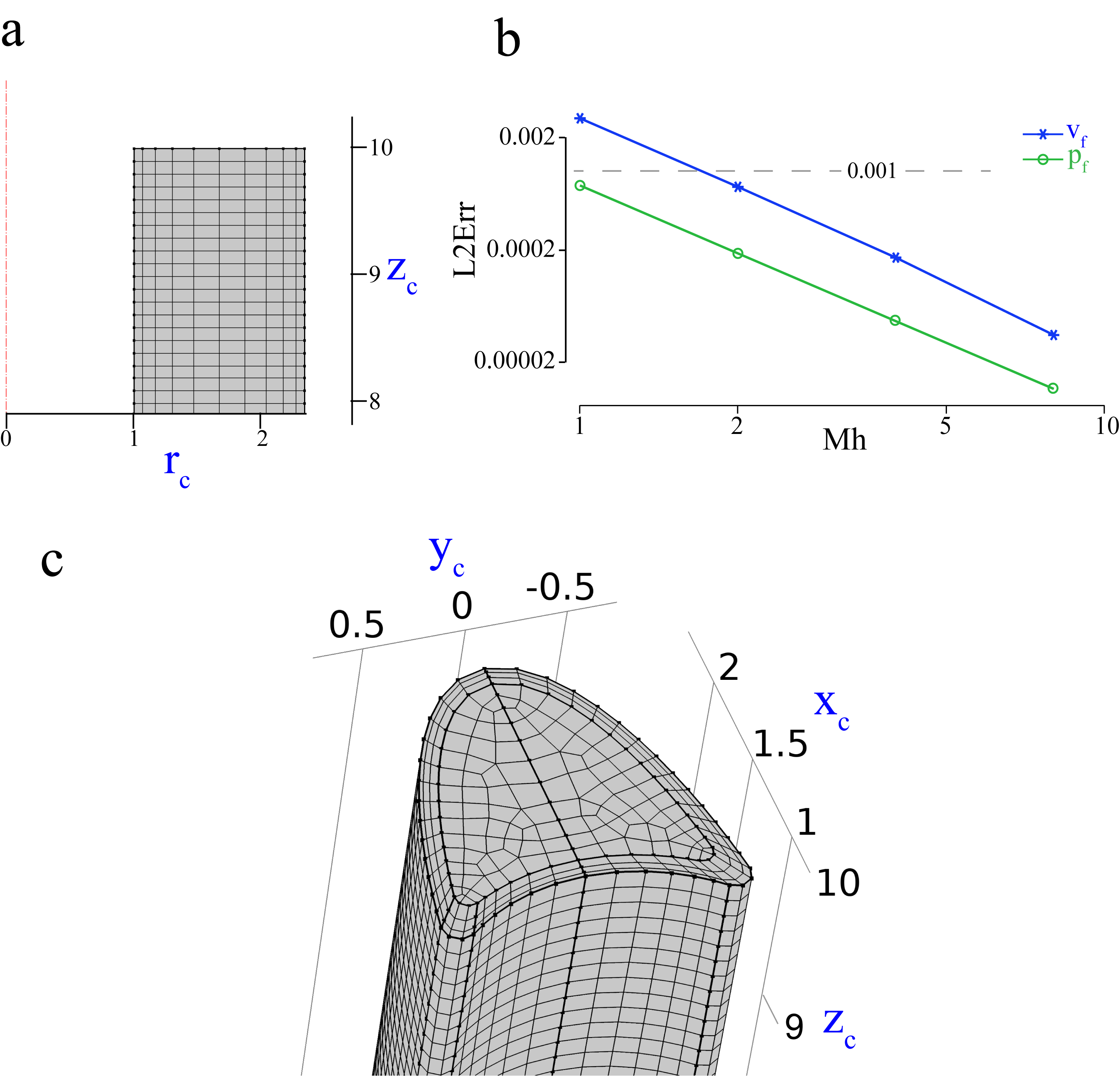

Supplement: Supplementary file 5 — Supplementary information5. [file 41598_2020_66887_MOESM5_ESM.tif]
