# Supplementary material for: Arterial pulsations drive oscillatory flow of CSF but not directional pumping
Source: Sci Rep. 2020 Jun 22;10:10102. doi: 10.1038/s41598-020-66887-w (PMC7308311; doi:10.1038/s41598-020-66887-w)
Supplement: Supplementary file 7 — Supplementary information7. [file 41598_2020_66887_MOESM7_ESM.pdf]

Supplementary material details for: Arterial pulsations drive oscillatory flow of CSF but not directional pumping

Authors: Ravi Teja Kedarasetti[1,2], Patrick J. Drew[1,2,3,4], Francesco Costanzo*[1,2,3,5]

Affiliations:

[1]Department of Engineering Science and Mechanics, The Pennsylvania State University, University Park, PA

[2]Center for Neural Engineering, The Pennsylvania State University, University Park, PA

[3]Department of Biomedical Engineering, The Pennsylvania State University, University Park, PA

[4]Department of Neurosurgery, The Pennsylvania State University, University Park, PA

[5]Department of Mathematics, The Pennsylvania State University, University Park, PA

* Correspondence and requests for materials should be addressed to Francesco Costanzo (fxc8@psu.edu)

1. **Supplement_002.docx –** Supplementary figures with description
2. **Appendix.pdf –** Appendix with full mathematical weak formulation used in finite element calculations
3. **SV1 –** Supplementary video1. Shows the movement of fluid driven by a simple 2D wall movement.
4. **SV2 –** Supplementary video2. Shows the movement of fluid driven by a periodic 2D wall movement.
